# Crystal Structure of the FeS Cluster–Containing Nucleotide Excision Repair Helicase XPD

**DOI:** 10.1371/journal.pbio.0060149

**Published:** 2008-06-24

**Authors:** Stefanie C Wolski, Jochen Kuper, Petra Hänzelmann, James J Truglio, Deborah L Croteau, Bennett Van Houten, Caroline Kisker

**Affiliations:** 1 Rudolf Virchow Center for Experimental Biomedicine, Institute for Structural Biology, University of Würzburg, Würzburg, Germany; 2 Department of Pharmacological Sciences, Stony Brook University, Stony Brook, New York, United States of America; 3 Laboratory of Molecular Genetics, National Institute of Environmental Health Sciences, National Institutes of Health, Research Triangle Park, North Carolina, United States of America; Brandeis University, United States of America

## Abstract

DNA damage recognition by the nucleotide excision repair pathway requires an initial step identifying helical distortions in the DNA and a proofreading step verifying the presence of a lesion. This proofreading step is accomplished in eukaryotes by the TFIIH complex. The critical damage recognition component of TFIIH is the XPD protein, a DNA helicase that unwinds DNA and identifies the damage. Here, we describe the crystal structure of an archaeal XPD protein with high sequence identity to the human XPD protein that reveals how the structural helicase framework is combined with additional elements for strand separation and DNA scanning. Two RecA-like helicase domains are complemented by a 4Fe4S cluster domain, which has been implicated in damage recognition, and an α-helical domain. The first helicase domain together with the helical and 4Fe4S-cluster–containing domains form a central hole with a diameter sufficient in size to allow passage of a single stranded DNA. Based on our results, we suggest a model of how DNA is bound to the XPD protein, and can rationalize several of the mutations in the human *XPD* gene that lead to one of three severe diseases, xeroderma pigmentosum, Cockayne syndrome, and trichothiodystrophy.

## Author Summary

Preserving the structural integrity of DNA, and hence the genetic information stored in this molecule, is essential for cellular survival. It is estimated that the DNA in each human cell acquires about 10^4^ lesions per day. Consequently, efficient DNA repair mechanisms have evolved to protect the genome. One of these DNA repair mechanisms, nucleotide excision repair (NER), is present in all organisms and is unique in its ability to repair a broad range of damage. In humans, NER is the major repair mechanism protecting DNA from damage induced by ultraviolet light. Defects in the genes and proteins responsible for NER can lead to one of three severe diseases: xeroderma pigmentosum, Cockayne syndrome, and trichothiodystrophy. The XPD protein is one of the key components of a ten-protein complex and is essential to initiate NER. In particular, the XPD protein verifies the presence of damage to the DNA and thereby allows DNA repair to proceed. We have solved the 3-dimensional structure of the XPD protein, and show how XPD has assembled several domains to form a donut-shaped molecule, which is able to separate two DNA strands and scan the DNA for damage. The structure also helps to explain why some of the mutations that have been identified in humans are associated with disease.

## Abstract 3

The structure of the DNA repair protein XPD provides insights into how the protein binds and recognizes damaged DNA and how mutations in*XPD* disrupt its function and lead to disease.

## Introduction

Nucleotide excision repair (NER) is the most versatile DNA repair pathway. [1–5]. NER is well known for its ability to remove bulky DNA lesions and is unique in its ability to repair structurally and chemically different substrates, including benzo[a]pyrene-guanine adducts caused by smoking, as well as guanine-cisplatin adducts formed during chemotherapy [6]. NER is the only repair mechanism in humans that is able to remove photoproducts induced by ultraviolet light. The phenotypic consequences of defective genes involved in NER are apparent in three severe diseases: xeroderma pigmentosum, Cockayne syndrome, and trichothiodystrophy [1,7–10]. The mechanism of the human NER system, while analogous to the well-characterized bacterial system, is less well understood. Over 30 proteins have been identified in humans that are critical for mediating the individual steps leading from damage recognition to incision and repair. However, due to the paucity of specific structural intermediates, the precise role for each protein has not been fully delineated.

NER has been proposed to proceed through either a “bipartite substrate discrimination” or a “multi-partite damage recognition” model [11,12]. It is generally believed that NER is initiated by the combined action of XPC and RAD23B, which recognize a general disruption of Watson-Crick base-pairing created in the vicinity of the damaged nucleotide. Both proteins are required to recruit the ten-subunit transcription factor TFIIH to this site. The XPD and XPB proteins are two helicases that are present in TFIIH, and which open the DNA around the lesion in an ATP-dependent fashion. This is the first catalytic step in this reaction pathway, leading to a conformational change that allows the recruitment of additional NER factors [5,13,14]. A second, more important function of the two helicases is damage verification. Recent data suggest very different roles for XPB and XPD [15]. The helicase activity of the XPB protein seems to be dispensable; however, its ATPase activity is essential for NER. This has been interpreted to suggest a wrapping of the DNA around XPB, which leads to an opening of the double-stranded DNA (dsDNA) close to the lesion. This opening allows the correct binding of XPD, which then utilizes its helicase activity to verify the damage and ensures that the backbone distortion is not the result of an unusual DNA sequence. This process was termed “enzymatic proofreading” and supports the bipartite damage recognition model in which the function of XPC-RAD23B is limited to the observation of a backbone distortion, and XPD is required to verify the damage through its helicase activity [16,17].

Very recently, it has been shown that the XPD protein contains an FeS cluster, which is essential for its function [18]. However, it is not clear whether the cluster has a structural role or is actively involved in the damage recognition process [19]. We solved the crystal structure of the XPD protein from Thermoplasma acidophilum, which shares high sequence identity to its eukaryotic homologs, and show that it contains two RecA-like helicase domains. The XPD protein displays high structural similarity to the bacterial UvrB protein, which is also required for enzymatic proofreading in NER. Two additional domains emerge from the first helicase domain and form a hole that is sufficient to allow passage of ssDNA. Furthermore, the structure delineates how different mutations in the protein lead to the human genetic disorders xeroderma pigmentosum, Cockayne syndrome, and trichothiodystrophy.

## Results/Discussion

### Overall Structure of XPD

Two different XPD-related protein sequences from T. acidophilum have been deposited in the National Center for Biotechnology Information (NCBI) and the Swiss-Prot databases, respectively. They differ only with respect to their N-terminus, with one of them containing 19 additional amino acids. We cloned both constructs and obtained crystals of the shorter protein, which was also active with respect to both its helicase and its ATPase activity (Figure S1). The protein crystallized in space group P6_5_ and the asymmetric unit contains one XPD molecule, indicating no higher oligomeric states, which is consistent with size-exclusion chromatography results and an analysis of the model using the PISA server [20]. The structure was solved by multiwavelength anomalous diffraction (MAD) using the anomalous Fe signal of the endogenous FeS cluster in the protein and was refined at 2.9 Å resolution to an *R*-factor of 0.209 and *R*_free_ of 0.287 (Table 1). The current model contains residues 23–507 and 515–615 (586 out of 602 residues) of the XPD construct with residues 20 to 22, 508 to 514, and 616 to 620 presumably being disordered.

**Table 1:**
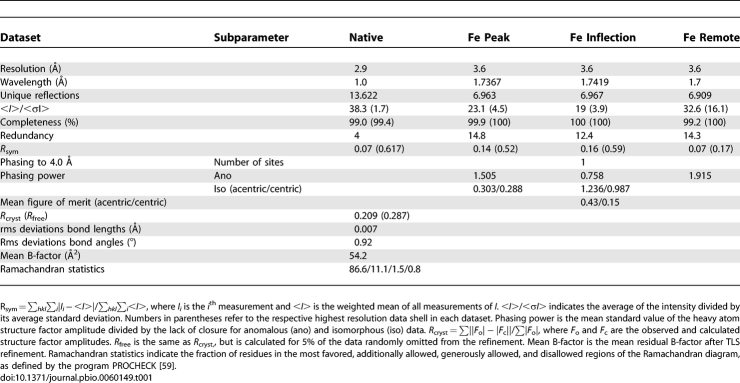
Crystallization, Data Collection, and Refinement Statistics

**Figure 1:**
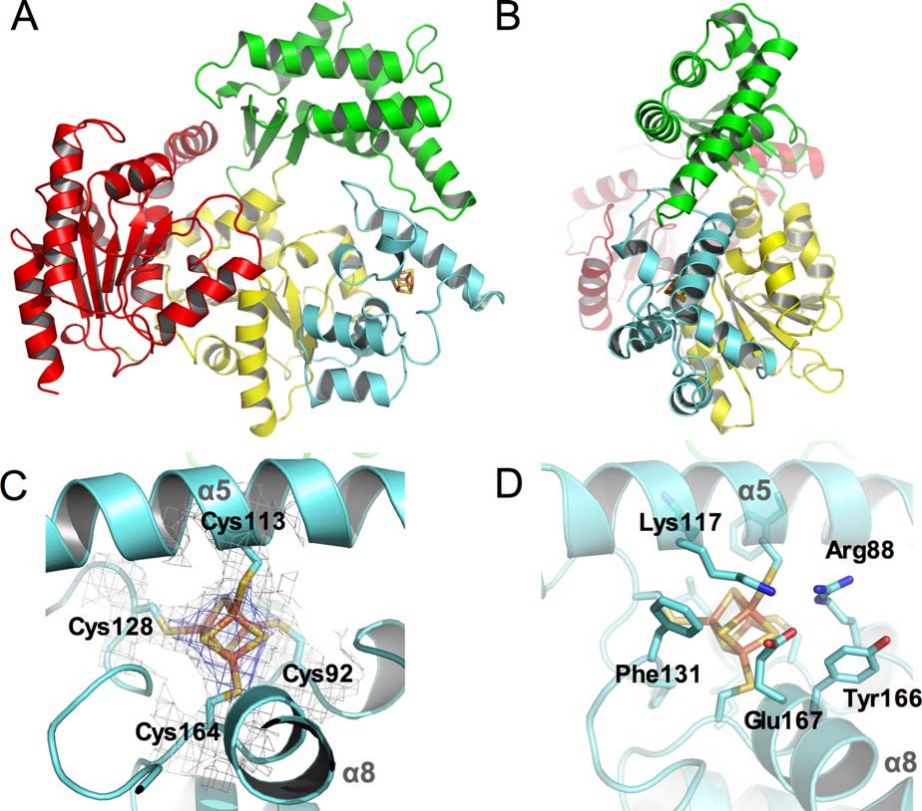
Overall Structure of XPD and the FeS Cluster (A) Front view of XPD, with domain 1 in yellow, domain 2 in cyan, domain 3 in green, and domain 4 in red. The FeS cluster is shown in all-bonds representation. (B) Side view of XPD color-coded as in (A). (C) SIGMAA weighted 2*F*_o_-*F*_c_ electron density map contoured at one times the standard deviation around the 4Fe4S cluster and the surrounding protein in grey, and at four times the standard deviation in blue. The cluster and the coordinating cysteines are shown in all-bonds representation. Secondary structure elements are labeled in grey. (D) Residues in close proximity to the 4Fe4S cluster. The cluster is shown as in (C), and residues Arg88, Tyr166, Lys117, Phe131, and Glu167 are shown in all-bonds representation, with α-helices and β-strands being labeled.

**Figure 2:**
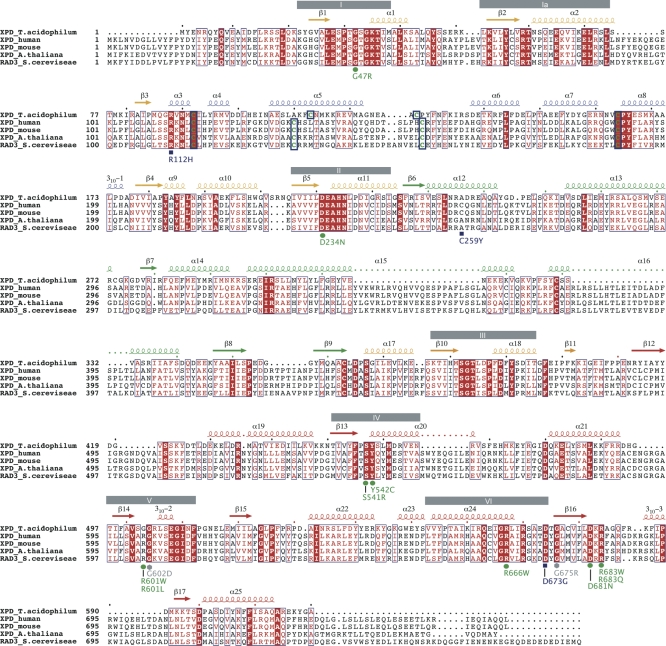
Sequence Alignment Sequence alignment of five different XPD proteins. From top to bottom: XPD from T. acidophilum, human, mouse, Arabidopsis thaliana, and Saccharomyces cerevisiae. Secondary structure elements are indicated above the sequence and are color coded according to their domains; arrows indicate β-strands and coils α-helices. Helicase motifs are shown as gray boxes. Mutations leading to xeroderma pigmentosum, Cockayne syndrome, or trichothiodystrophy are indicated below the sequence and are colored in blue, green, and grey, referring to their occurrence in trichothiodystrophy, xeroderma pigmentosum, or xeroderma pigmentosum/Cockayne syndrome patients, respectively. Conserved residues are boxed, and strictly conserved residues are shown in white with a red background. The four cysteines that coordinate the 4Fe4S cluster are highlighted in green surrounded by thick blue boxes.

The structure of the protein can be divided into four distinct domains. Domain 1 is formed by residues 23–87, 178–225, and 366–407, domain 2 by residues 88–177, domain 3 by residues 226–365, and domain 4 by residues 408–615 (Figure 1A and 1B). The first three domains together with α-helix 22 from domain 4 form a donut-shaped structure containing a hole with a diameter of approximately 13 Å (Figure 1A). The remainder of domain 4 is positioned in front of the ring without obstructing the hole of the donut. The overall dimensions of the protein can therefore be divided into the donut with a width and height of 65 Å and 75 Å and a thickness of 29 Å. At the location of domain 4, the width of the ring is increased to 45 Å (Figure 1A and 1B). Domains 1 and 4 represent the “classical” RecA-like fold that is present in all helicases of superfamilies 1 and 2 (SF1 and SF2) [21]. Both domains share approximately 9% sequence identity and can be superimposed with a root mean square (rms) deviation of 2.4 Å using 101 Cα-atoms out of 153 from domain 1, and 201 from domain 4, respectively. Both domains display a similar α/β/α sandwich architecture with a central parallel seven-stranded β-sheet surrounded by seven α-helices in domain 1 and a six-stranded β-sheet surrounded by seven α-helices and two 3_10_ helices in domain 4. The interface between domains 1 and 4 forms the composite ATP binding site. Domain 1 contains helicase motifs I, Ia, II, and III, whereas domain 4 harbors helicase motifs IV, V, and VI [22] (Figure 2). In the context of the overall XPD structure, domain 1 can be viewed as the core domain surrounded by the other three domains. Domains 2 and 3 are insertions, which emerge from domain 1. Domain 2 is inserted between β-strands β3 and β4, while domain 3 is inserted between α-helices α11 and α17. Domain 4 is situated adjacent to domain 1 within the linear protein sequence (Figures 1 and 2).

Notably, the closest related homolog of the full-length XPD structure as revealed by similarity searches [23] was UvrB [24], which has been proposed to be the prokaryotic equivalent to XPD and utilizes its helicase activity for damage verification. XPD and UvrB can be superimposed with an rms deviation of 2.6 Å using 254 aligned Cα atoms out of 588 and 505 residues, respectively. The match is mostly mediated via the two helicase domains, whereas the other domains have no significant structural similarity to each other (Figure S2). In addition, we compared XPD to Hel308 and NS3, two SF2 helicases (Figure S3). The superposition shows that structural similarities are again mainly confined to the RecA domains, whereas the auxiliary domains are highly variable. Hel308 and NS3 have been structurally characterized with DNA substrates, and both represent a closed state of the helicase framework [25,26]. No adenosine nucleotide is bound in these structures, but they are presumed to be in a preprocessive state that only requires ATP binding to reach the processive state [25]. Using the first RecA domain (domain 1) as a reference point for superposition with either Hel308 or NS3, XPD assumes a more open state that is mainly mediated via a rotation of the second RecA domain (domain 4) of about 30° or 16°, respectively, relative to domain 1 (Figure S3C). The composite ATP binding site is located near the hinge region when compared to the closed state of the other two helicases. Our structure may therefore reflect a ground state of XPD prior to nucleotide and/or DNA binding that underlines the conformational flexibility necessary to translate chemical energy into motion.

### The FeS Cluster Domain

The first insertion into helicase domain 1 is of particular interest since it contains an FeS cluster, a unique feature among the XPD-like SF2 helicases [18]. Domain 2 displays an exclusively α-helical architecture consisting of six α-helices and one 3_10_ helix that surround the central 4Fe4S cluster (Figure 1A, 1C, and 1D). The FeS cluster is coordinated by four cysteines, consistent with the coordination typically observed in 4Fe4S clusters, and all four cysteines display continuous connectivity in the electron density maps (Figure 1C). A comparison of the B-factors between the 4Fe4S cluster and the surrounding protein residues reveals similar values, indicating full occupancy of the cluster. Three of the coordinating cysteines (Cys92, Cys128, and Cys164) are located in loops, whereas the fourth cysteine, Cys113, is located in a central position within α-helix 5 (Figures 1 and 2). Surprisingly, it was shown that the helicase activity is not affected when Cys102 or Cys105 in Sulfolobus acidocaldarius or Ferroplasma acidarmanus XPD, respectively, were mutated to serine [18,19]. These two residues correspond to Cys113 in our structure. Pugh et al. [19] suggested that the aerobically purified protein most likely contained a degraded 3Fe4S cluster, which is still functional, but presumably a 4Fe4S cluster is present in vivo. When any of the remaining cysteines is mutated to serine, however, the helicase activity of the enzyme is abrogated [18,19].

**Figure 3:**
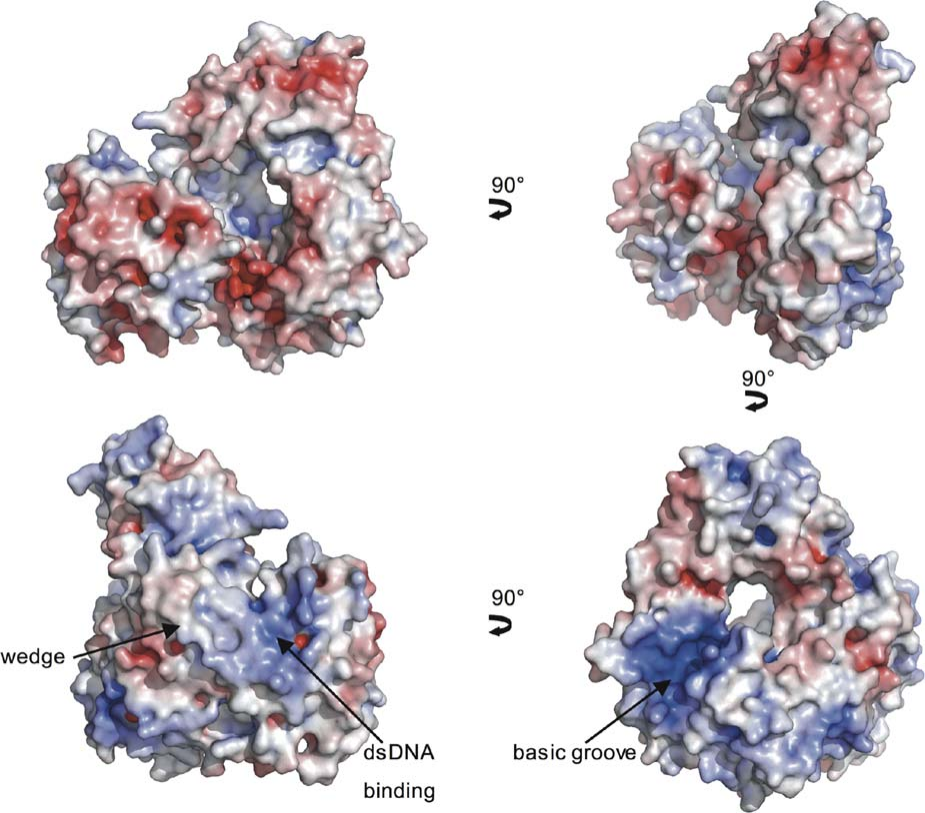
Electrostatic Surface Potential of XPD Four different views of the electrostatic surface potential of XPD. The surface potential has been calculated with Pymol/APBS at an ionic strength of 150 mM and is contoured at ±10 *k_B_T*. The first view is the same as shown in Figure 1A; the arrows show the transition from one view to the other with the rotation indicated by the arrow. Putative DNA binding sites are marked by additional arrows.

The cluster is further stabilized predominantly by hydrophobic interactions. Residues Arg88 and Tyr166, which shield the cluster from solvent exposure, are strictly conserved and face towards a pronounced solvent-exposed groove that is formed by α-helices 5 and 8 from domain 2 and α-helix 10 from domain 1 at the back of the protein (Figures 1D and 3). The closest structural homolog for this domain identified by a secondary structure matching search [23] revealed c-myb, a transcription factor that does not contain an FeS cluster [27]. Although c-myb superimposes with a relatively low Q-score of 0.15 (Figure S2B), it is notable that the structural similarity is restricted to the DNA binding interface of c-myb. c-Myb superimposes well with α-helices 5, 6, 7, and 8 of domain 2, of which helices 5 and 8 coincide with the DNA binding interface of c-myb (Figure S2B). In the XPD structure, these helices form part of the groove mentioned above, thus indicating a possible DNA binding site. This is further emphasized by the basic nature of this groove (Figure 3), which is composed of several highly conserved, positively charged residues. However, no significant sequence conservation can be identified between c-myb and XPD in the structurally homologous regions.

### The α-Helical Domain

Domain 3 consists mostly of extended α-helices (α-helices 12, 13, 14, 15, and 16) and four additional antiparallel β-strands (β6, β7, β8, β9) building a “β-bridge” to domain 1. The β-bridge is further stabilized by α22, an α-helical extension located between β15 and α23 of domain 4. The helices can be grouped into two α-helical hairpins that stack with each other, with one hairpin containing α12 and α13, and the second containing α15 and α16, which is slightly distorted by the insertion of a loop. The two helical hairpins intersect at an angle of approximately 60° and create an extensive hydrophobic core between them. Helix α14 is situated in the V-shaped opening that is formed by the tilt between the two α-helical bundles (Figure 1A and 1B). Similarity searches revealed no significant hit, indicating that this fold has not been encountered previously.

The ring of the donut is closed at its thinnest side via an interface between domains 2 and 3 that has a buried surface area of approximately 620 Å^2^. The interface is formed by 17 residues from each domain, which display little sequence conservation apart from Phe326, which is always an aromatic residue (Figure 2). Most of the interactions are hydrophobic in character, additionally four salt bridges can be observed between Lys323/Asp99, Arg335/Glu103, Arg235/Glu103, and Glu315/Lys111.

### The Role of the FeS Cluster

**Figure 4:**
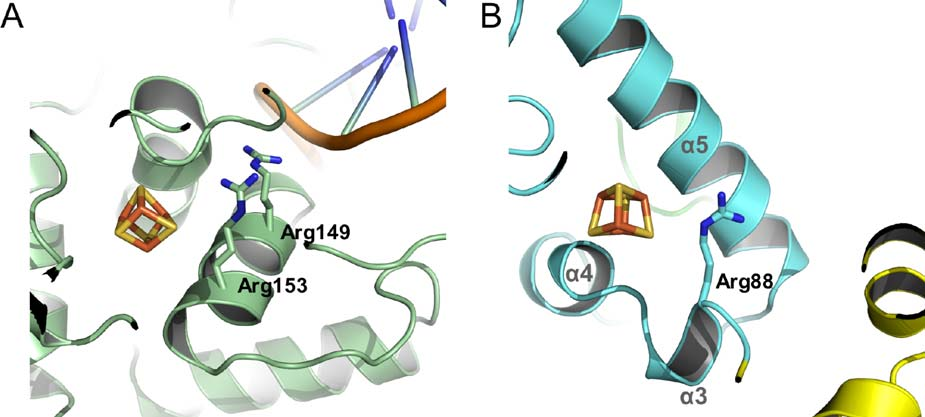
Structural Similarity to MutY Side-by-side presentation of the 4Fe4S clusters in MutY and XPD after superposition (A). Two arginines (Arg149 and Arg 153) are located in close proximity to the 4Fe4S cluster and point towards the DNA in MutY (B). In XPD, Arg88 occupies a similar position as observed for the arginines in MutY. The helices surrounding the cluster have been labeled.

Since the presence of the FeS cluster is essential for helicase activity on dsDNA [18,19], it prompted us to investigate the only other structurally characterized DNA-binding proteins with such a feature, the base excision repair proteins, MutY and Endo III [28,29], with a focus on the first because a structure of a MutY-DNA complex has been described [28]. For MutY, it was shown that its FeS cluster is required for enzymatic activity and DNA binding [30]. The XPD protein contains a loop motif in the FeS cluster domain with a high density of positively charged residues similar to the FeS cluster loop motif (FCL) in MutY [31]. The superposition of the XPD and MutY FeS cluster domains (Figure 4) reveals a similar orientation of two conserved arginines (Arg88 in XPD and Arg153 in MutY). In MutY, it was shown that a neighboring conserved arginine, Arg149, is perfectly positioned for an interaction with the DNA backbone, and bridges the distance between Arg153 and the DNA [32]. Based on the similarity to MutY, Arg88 in XPD may fulfill a similar function. Furthermore, the position of Arg88 at the surface of a pocket where DNA recognition could take place supports the idea proposed by Lukianova et al. that the FeS cluster plays an important role in arranging the residues of the FCL motif for DNA binding [31].

For MutY, it was shown that the redox properties of the [4Fe-4S]^2+^ cluster are modulated by the presence of DNA [33]. DNA-binding activates the cluster and facilitates oxidation [34]. Boal et al. proposed a model for DNA-mediated charge transfer (CT) in DNA repair in which one electron is transferred from the cluster to the DNA. In this model, the CT acts as an initial sorting mechanism, enabling a rapid scanning of undamaged regions by several glycosylase molecules, so that they are able to relocate themselves onto sites near the damage [34]. In NER, an analogous scanning mechanism seems unlikely, but a change in oxidation state of the 4Fe4S cluster upon DNA binding and as part of the damage verification step may be required, thus suggesting a functional role for the 4Fe4S cluster and not just a structural role. This hypothesis is further supported by site-directed mutagenesis studies that demonstrate that single mutations of three of the four 4Fe4S cluster coordinating cysteines to serine lead to a loss of the 4Fe4S cluster, and abrogate helicase activity, but retain a correctly folded protein that is still able to translate along ssDNA [18,19].

### DNA Binding and Unwinding

**Figure 5:**
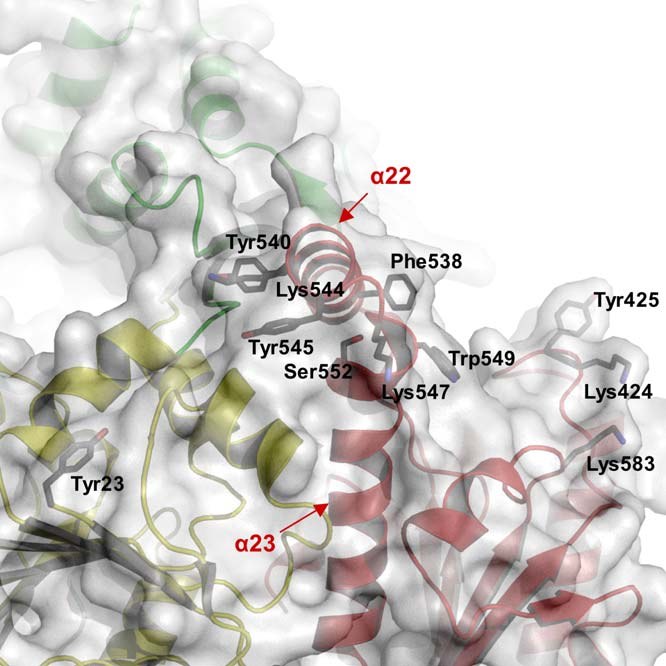
The Wedge A view towards helices α22 and α23 in domain 3. The color code from Figure 1A was maintained, and a transparent surface was added to provide a view of the groove where one DNA strand binds. All residues that may play a role in double strand separation are indicated and labeled. Helices α22 and α23 have been labeled and indicated by arrows.

**Figure 6:**
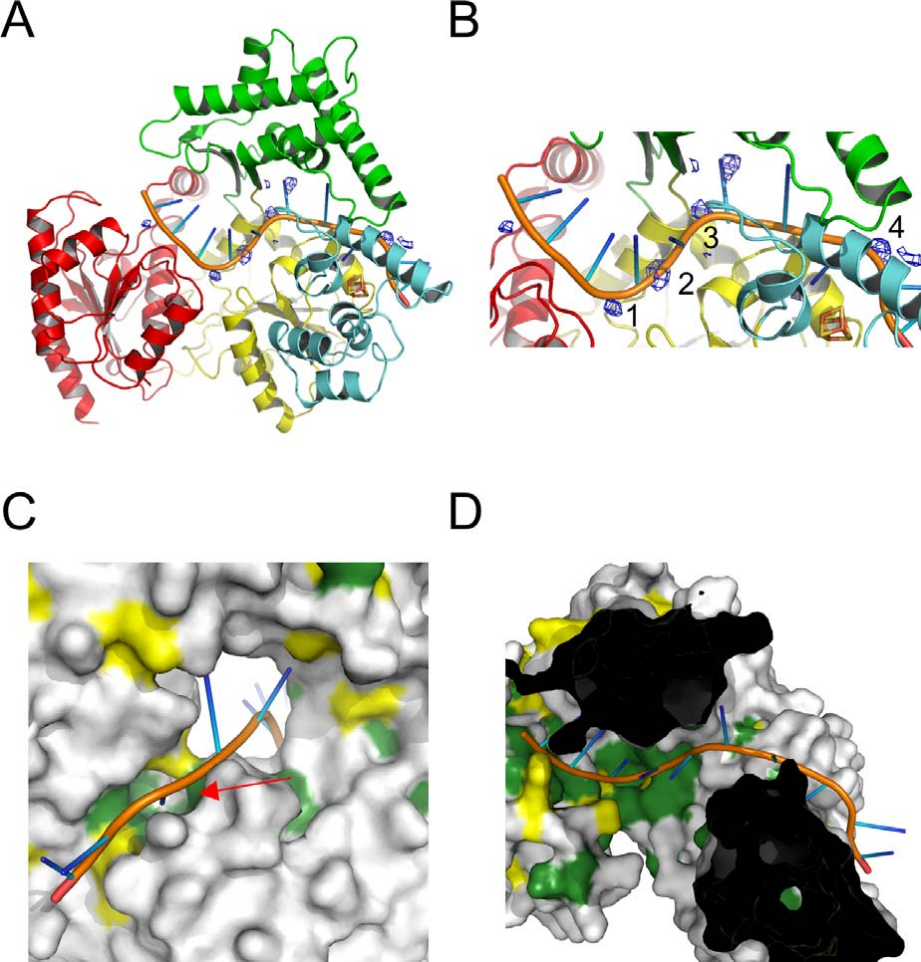
Hypothetical XPD-DNA Model (A) Overall view of XPD in complex with ssDNA. The model was obtained by superposition with the NS3 helicase in complex with ssDNA [26]. Residual difference map peaks at 2.5 times the standard deviation are shown in blue, and peaks used for backbone phosphate positioning are numbered. Further extension of the ssDNA towards the hole of XPD was achieved by the addition of B-form DNA. The DNA is shown with its backbone in orange, and the bases are shown as blue spokes. (B) Close-up view from (A). (C) Surface representation of the XPD–DNA model. The DNA is shown as in (A), and the surface is colored according to sequence conservation, with green indicating strictly conserved, yellow highly conserved, and gray residues that are not conserved. The DNA in this view emerges from the hole and fits nicely into the highly conserved pocket (indicated by the red arrow), which could potentially play a role in damage recognition or which couples DNA binding to the FeS cluster. (D) Top view of the XPD-DNA model. For clarity, the top part of XPD has been removed to allow a view into the highly conserved groove, which leads the ssDNA towards the hole in the donut-shaped molecule.

The XPD protein is a member of the SF2 helicases. To obtain insight into the DNA binding mode of XPD, we calculated the electrostatic surface potential of the protein and searched for conserved solvent-exposed amino acids (Figures 2, 3, 5, and 6). The surface potential indicates a positively charged path for dsDNA along domain 4, leading towards a highly conserved groove along domain 4 and domain 1, which provides sufficient space for ssDNA and directs the DNA towards the hole formed by domains 1–3. The dsDNA requires separation into ssDNA prior to entering the groove. Recently, the structure of the SF2 helicase Hel308 was determined in complex with DNA, and a prominent β-hairpin in the second helicase domain was identified that is responsible for initial strand separation [25]. It was proposed that this β-hairpin could be a general feature of SF2 helicases. In XPD however, this “wedge” is formed more likely by an α-helical extension in domain 4 (Figure 5). Despite the difference in secondary structure, it is located between helicase motifs V and VI as demonstrated for Hel308 and proposed for NS3 [25] (Figure S3). Two α-helices in XPD, α22 and α23, form two walls of the wedge and extend farther out towards the solvent compared to other helicases such as UvrD and PcrA [35,36].

We propose that the tip of the wedge composed of residues in the loop between α22 and α23 separates the two DNA strands. The last two turns of α22 and the first two helical turns of α23 contain several aromatic amino acids, which could stabilize the separated DNA strands in a fashion similar to that observed for Hel308. On one side of this wedge, the highly conserved residues Tyr540 and Tyr545 are oriented with their side chains pointing towards the solvent where they could easily form stacking interactions with the exposed bases of ssDNA. These stacking interactions can then be continued by additional solvent-exposed aromatic residues, such as Tyr23, leading the ssDNA along the back of the protein to a position where the two strands meet again to reform dsDNA. Although exposed aromatic residues are also present on the other side of the wedge, their degree of conservation is relatively low. In our structure, Phe538 and Tyr425 could both stack against the bases in ssDNA. However, only Tyr425 is conserved, whereas Phe538 is replaced by a leucine in eukaryotic XPDs. This substitution appears to be compensated by the occurrence of Phe651 in human XPD, which substitutes for Ser552 in T. acidophilum XPD; and due to the close spatial proximity of the two side chains, they would assume similar positions (Figure 5). Consequently, there is one phenylalanine available that would represent the required stacking partner. In addition, several highly conserved, positively charged residues, such as Lys583 and Lys424, apparently define the path for the second strand leading into the groove described above and from there continues through the central hole (Figures 5 and 6).

Despite the fact that we crystallized the protein in the absence of DNA and phosphate buffer, we identified significant peaks with heights of more than 2.5 times the rms deviation in difference electron density maps (Figure 6) that are spaced by approximately 6.5 Å, as well as slightly longer distances and cannot be explained by the protein model. Since the distance between phosphates in ssDNA is approximately 6.4 Å, it is therefore very tempting to speculate that some DNA remains bound to the protein during purification and gives rise to these residual electron density features. Further support for this hypothesis is provided by the superposition of our structure with NS3 helicase in complex with ssDNA [26] (Figure S3). Based on this superposition, we have built a model for a ssDNA binding mode (Figure 6) in which the extension of the ssDNA towards the hole positions three of the phosphates into the residual electron density peaks.

The postulated DNA route passes by another highly conserved surface feature in XPD, a narrow pocket that is formed by the strictly conserved Arg88 and Tyr166 on one side and Tyr185 on the other side, and is located in the wall of the central hole, directly adjacent to the 4Fe4S cluster (Figure 6). The dimensions and shape of this pocket are ideally suited to accommodate a nonmodified purine or pyrimidine base, which would be held in place through van der Waals interactions with the residues mentioned above. Due to its location, this surface feature would allow a direct coupling between the FeS cluster and a readout of the DNA. This pocket is reminiscent of the pocket for the flipped-out base that was observed in the UvrB-DNA structure [24] .

### Damage Recognition

Initial DNA distortion recognition in eukaryotes is achieved through the combined action of XPC and RAD23B [37]. It was shown that with the recruitment of TFIIH to the site of damage, the helicase XPD is required for proofreading, whereas XPB fulfills a structural role [15]. In the absence of an XPD-DNA complex containing a lesion, the process of proofreading remains highly speculative. The structure of XPD clearly reveals structural homology to its prokaryotic homolog UvrB. In UvrB, it was shown that a β-hairpin, which emerges from the first helicase domain, is critical for damage recognition [38–40]. However, despite the structural similarity between the two proteins, XPD does not contain a corresponding feature. In our model of the XPD-DNA complex (Figure 6), we propose that one of the DNA strands passes through the central hole, which is formed by domains 1–3. According to studies by Naegeli et al. [41], this would be the translocating strand, which contains the lesion, and leads to a stalled protein-DNA complex. The dimension of this hole, with a diameter of 13 Å, however, is most likely too big to provide a trap for damaged DNA. One possible candidate for the “analysis” of each base with respect to their correct structure would be the narrow pocket in the wall of the central hole described above. The size of this pocket suggests that only nondamaged bases could be accommodated, whereas a bulky DNA substrate would be excluded. This pocket is also an attractive candidate for the damage recognition process due to its close proximity to the 4Fe4S cluster and the involvement of Arg112 of human XPD (Arg88 in our structure), which has been shown to cause trichothiodystrophy when mutated to histidine.

### Mutations Leading to Xeroderma Pigmentosum, Cockayne Syndrome, or Trichothiodystrophy

**Figure 7:**
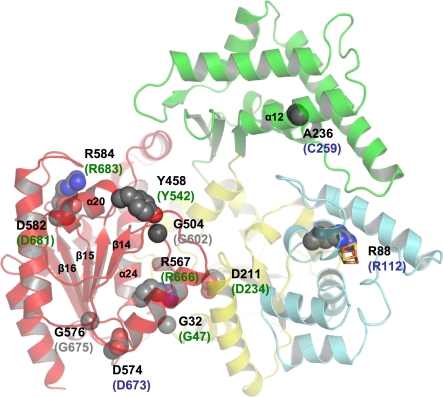
Mutants Leading to Xeroderma Pigmentosum, Cockayne Syndrome, or Trichothiodystrophy XPD is shown in ribbon presentation with the same orientation and color code for the different domains as in Figure 1A. Point mutations leading to either xeroderma pigmentosum, Cockayne syndrome, or trichothiodystrophy are shown in a space-filling representation and are labeled according to their residue number for T. acidophilum XPD in black and, according to the disease they cause in humans, green (XP), blue (TTD), and grey (XP/CS). Secondary structure elements mentioned in the text are labeled.

TFIIH in humans is not only required for DNA repair, but is also essential for transcription [42]. XPD represents one of the ten protein subunits of TFIIH and interacts tightly with the N-terminal 236 amino acids of p44. This interaction results in a 10-fold increase in its helicase activity [43]. It has been shown that the helicase function of XPD is not required for transcription, but is essential for NER [44]. On the other hand, XPD is required to stabilize the interaction between the core TFIIH complex, which contains seven subunits, and the cdk-activating kinase (CAK) subcomplex, consisting of the remaining three subunits [45,46]. Mutations in XPD (Figure 2 and 7) can therefore lead to three different effects. The first class of mutations affects the activity of the protein directly, whereas the second group can lead to impaired interactions with p44, thus affecting its own activity in an indirect way. The third group of mutations may lead to a destabilization of TFIIH, thereby reducing overall transcriptional activity. Based on our structure the effects of several point mutations leading to xeroderma pigmentosum, Cockayne syndrome, or trichothiodystrophy can be explained (Figure 7). Point mutations associated with xeroderma pigmentosum, such as G47R, D234N, and R666W, are located in helicase motifs I, II, and VI, respectively, and impair the ability to bind and hydrolyze ATP, thus inactivating the enzyme; however, point mutations within other regions have quite distinct effects.

Arg112 (Arg88 in T. acidophilum) is located in the FeS cluster domain and is in direct contact with the cluster. A mutation of this residue to histidine has been identified in several TTD patients [47]. Analysis of the equivalent residue in S. acidocaldarius XPD abolished its helicase activity [18]. Arg88 is located in close vicinity to Cys113 one of the Fe-ligands, and shields the cluster, with its long side chain, from solvent. It is the first residue in a short α-helix, α 3, which together with the opposite side of the helix forms one wall of the hole where ssDNA most likely passes through (Figure 6). The proposed role for Arg88 in analogy to MutY as described above may be accomplished by Arg112 in the human XPD protein and a mutation to histidine, as observed in trichothiodystrophy patients, could prevent this interaction, thus reducing the affinity of the protein to the DNA. However, the exact role of the 4Fe4S cluster, whether it is involved directly in the recognition process or the translocation along the DNA, remains speculative at this point. It is interesting to note that Egly and coworkers have shown this variant in human XPD to be completely devoid of helicase activity [48].

The effects of the C259Y variant can also be readily explained. This cysteine is replaced in T. acidophilum by another small hydrophobic residue, Ala236, in α-helix 12, which points into the hydrophobic core within domain 3. This core stabilizes the relative position of the four α-helices within this domain as outlined above. Replacing this small hydrophobic residue with a tyrosine leads to severe steric clashes within this core and thereby destabilizes the entire domain.

The two mutants Y542C and G602D are very close to each other in the structure. Tyr458 (Tyr542 in human XPD) is located at the beginning of α-helix 20 in domain 4 and forms hydrophobic interactions with another strictly conserved residue, Val501 (Val599 in human XPD), in a neighboring β-strand. Replacing the tyrosine with a cysteine would weaken the interactions between this helix-strand pair. Gly504 (Gly602 in the human enzyme) is positioned between β-strands 14 and 15 in domain 4. If this residue were to be replaced by a larger residue, it would point towards Tyr458 (Tyr542 in human XPD) and would thereby interfere with this side chain.

The remaining four mutations D673G, G675R, D681N, and R683W/Q, although causing different diseases, are all clustered closely together towards the C-terminal end of the human XPD protein and correspond to residues Asp574, Gly576, Asp582, and Arg584 in *T. acidophilum XPD*, respectively. It has been speculated that residues at the C-terminal end of human XPD interfere with p44 binding, thus leading to an inability to stimulate the helicase activity of XPD [43]. Of these four mutations, only G675R was analyzed with respect to its ability to interact with p44, and it was shown that the interaction was severely diminished [43]. All other analyzed disease mutants are located further towards the C-terminal end of human XPD where our archaeal XPD contains no corresponding residues, which is not unexpected since T. acidophilum does not contain a p44 homolog. T. acidophilum Asp574, Gly576, Asp582, and Arg584 are located in domain 4 and fulfill important structural roles. Asp574 forms interactions with the strictly conserved Arg570 (Arg669 in the human enzyme), which is located at the end of helix 24, and thereby stabilizes the transition from the helix to the following β-strand 16. Gly576 is positioned in this β-strand and points towards two hydrophobic residues, Leu568 and Ile569 (Ala667 and Ile668 in human XPD) in α24. A mutation of Gly675 to an arginine would push the entire helix away from the β-strand and thereby destabilize the integrity of the domain. Asp582 is located directly behind β16 and forms tight interactions with the strictly conserved Arg584 (Arg683 in human XPD), and the latter forms additional interactions with Asp426 and Phe527 (Glu509 and Tyr625 in human XPD), two highly conserved residues. The point mutations at the C-terminal end of XPD thus clearly play important structural roles, and any of the four mutations would interfere with the fold of domain 4, which could also diminish the interactions with p44. According to our protein–DNA model, however, T. acidophilum Arg584 (Arg683 in human XPD) also plays an important role in DNA binding and is one of the residues that may bind to the DNA close to the double-strand/single-strand junction. Replacing this positively charged residue with either a glutamine or tryptophan may severely interfere with DNA binding and thereby lead to the disease phenotype.

The crystal structure of XPD from T. acidophilum revealed that the protein contains two RecA-like helicase domains and two additional domains that emerge from the first helicase domain. Surprisingly, the first three domains form a donut-shaped structure and a protein–DNA model is proposed in which one of the ssDNA strands passes through this central hole in close spatial proximity to the 4Fe4S cluster in the second domain. The high sequence homology to eukaryotic XPDs allowed the analysis of mutations leading to one of the three severe diseases xeroderma pigmentosum, Cockayne syndrome, or trichothiodystrophy and provides the basis for a more detailed analysis to understand the combined action of the helicase and the 4Fe4S cluster to achieve damage verification within the NER repair cascade.

## Materials and Methods

### Protein expression and purification.

The genes encoding two XPDs from T. acidophilum with variable N-termini (residues 1–622 and 23–622) were cloned into the pET16b vector (Novagen) using the NdeI and XhoI restriction sites. XPD was expressed as an N-terminally His-tagged protein in Escherichia coli BL21-CodonPlus (DE3)-RIL cells (Stratagene) by induction with 0.1 mM isopropyl-β-thiogalactoside at 14 °C for 18 h. The protein was purified by metal affinity chromatography (Ni-NTA; Invitrogen) followed by size-exclusion chromatography (HiLoad 26/60 Superdex 200 prep grade; GE Healthcare) in 20 mM Tris (pH 8) and 500 mM NaCl. The protein was concentrated to 5 mg/ml based on a molar absorption coefficient of 65,140 M^−1^ cm^−1^.

### Helicase assay.

For construction of the 5′ overhang DNA substrate, a 25-mer oligonucleotide (MDJ1, 5′-GACTACGTACTGTTACGGCTCCATC-3′) was 5' end labeled and annealed to the 3' end of a 50-mer oligonucleotide (NDB, GCAGATCTGGCCTGATTGCGGTAGAGATGGAGCCGTAACAGTACGTAGTC). The helicase assay was carried out as described by [18] with slight modifications. Briefly, the reactions (10 μl) were incubated at room temperature in 20 mM MES (pH 6.5), 1 mM DTT, 0.1 mg/ml BSA, 5 mM MgCl_2_, 10 nM ^32^P-labeled DNA substrate, and 500 nM XPD for 10 min. The reactions were started by the addition of 3 mM ATP and transferred to a 45 °C water bath. After the specified time, 20 μl of stop solution (10 mM Tris-HCl [pH8]. 5 mM EDTA, 5 μM cold competitor [MDJ1], 0.5% SDS, and 1 mg/ml proteinase K) was added and incubated for 15 min at 37 °C to allow proteinase K digestion. Samples were separated on a native 10% acrylamide:bis TBE gel for 1 h at 100 V.

### Crystallization, data collection, and structure solution.

XPD crystals were grown by vapor diffusion in hanging drops containing equal volumes of protein in 20 mM Tris/HCl (pH 8.0) and 500 mM NaCl at a concentration of 5 mg/ml, and a reservoir solution consisting of 200 mM MgCl_2_, 100 mM Hepes (pH 8), and 5%–10% PEG 400 equilibrated against the reservoir solution. Crystals grew within 7 d at 20 °C to a maximum size of 100 × 50 × 50 μm^3^. Prior to data collection, the crystals were cryocooled by sequential transfer into mother liquor containing increasing amounts of glycerol in 5% steps to a final concentration of 30%.

The crystals were flash cooled in liquid nitrogen, and data collection was performed at 100 K. Data sets were collected at beamline BM14 (European Synchrotron Radiation Facility [ESRF]) at wavelengths of 1.0 Å, 1.7 Å, 1.7367 Å, and 1.7419 Å. All data were indexed and processed using Moslfm and Scala [49,50]. The crystals belong to space group P6_5_ with unit cell dimensions of *a* = *b* = 78.9 Å, *c* = 174.0 Å. Structure solution was achieved utilizing the anomalous signal of the endogenous Fe belonging to the 4Fe4S cluster by MAD data collection at the Fe edge. The peak and inflection datasets were obtained from one crystal and were merged with a highly isomorphous dataset collected at the remote wavelength. The Fe sites were located using ShelxD [51], and phase improvement was achieved with Sharp [52]. Substructure solution and refinement was carried out at 4 Å resolution, and the 4Fe4S cluster was treated as a “super” atom for phasing. The initial maps were subjected to solvent flattening and phase extension to 3.6 Å using the programs Solomon [53] and Pirate [54]. The solvent-flattened maps were autotraced using the low-resolution quick-build option in ARP/WARP [55] and further extended manually using the programs O and Coot [56,57]. After assigning the maximum number of residues and side chains possible, the model was subjected to phase-restrained simulated annealing and maximum likelihood refinement using the program phenix.refine [58]. Refinement was carried out against the highest resolution dataset up to 2.9 Å. The model was further improved by alternating rounds of refinement and manual model building. When the model was sufficiently complete, refinement continued with TLS and restraint maximum likelihood refinement using Refmac5 [54]. The final model contains 586 out of 602 amino acid residues, the 4Fe-4S cluster, one calcium ion, and one water molecule.

## Supporting Information

Figure S1 Helicase Activity of T. acidophilum XPD (A) Graphic representation of helicase assay.
(B) XPD is an ATP-dependent DNA helicase. Lane 1 is the ssDNA control, lane 2 dsDNA without XPD, lanes 3–6 contain 500 nM XPD in the absence (lanes 3 and 4) of ATP and in the presence of ATP (lanes 5 and 6). The 5-min and 15-min incubation times were analyzed and are shown in lanes 3 and 5 or 4 and 6, respectively.
(322 KB PDF)

Figure S2 Superposition of XPD with UvrB and with cMyb (A) XPD, color coded as in Figure 1A, was superimposed with UvrB shown in grey. The two RecA-like domains (yellow and red) superimpose well, whereas the remainder of the two protein models share no structural homology.
(B) Superposition of the 4Fe4S cluster containing domain of XPD with cMyb. XPD is shown in cyan and cMyb in grey.
(959 KB PDF)

Figure S3 Superposition of XPD with Hel308 and NS3 Helicases (A) XPD is color coded as in Figure 1A; Hel 308 is shown in light blue. Main chain atoms of the wedge in XPD and the β-hairpin in Hel308 are shown in space-filling models and are indicated by arrows. The DNA is shown in grey, and the bases as grey spokes. The superposition of XPD with Hel308 revealed an rms deviation of 3.6 Å using 240 Cα-atoms out of 683 from Hel308 and 588 from XPD.
(B) XPD is color coded as in (A), and NS3 is shown in blue. The superposition of NS3 helicase with XPD led to an rms deviation of 3.0 Å using 205 Cα-atoms out of 432 from NS3 and 588 from XPD.
(C) Superpositions of the RecA-like domains of XPD (yellow and red), NS3 (blue), and Hel308 (light blue) using the first RecA domain (domain 1) as the pivot point.
(1.59 MB PDF)

### Accession Numbers

Coordinates and structure factors for the XPD structure have been deposited in the Protein Data Bank (http://www.rcsb.org/pdb/home/home.do) using the Autodep tool from the European Bioinformatics Institute (http://www.ebi.ac.uk/) with the entry code 2VSF.

The Protein Data Bank accession numbers for the proteins discussed in the paper are as follow: c-myb (1h89), UvrB (2fdc), Hel308 (2p6r), and NS3 (1a1v).

## Abbreviations

dsDNA: double-stranded DNA
NER: nucleotide excision repair
rms: root mean square
SF: super family
ssDNA: single-stranded DNA
