# Supplementary figures and images for: Crystal Structure of the FeS Cluster–Containing Nucleotide Excision Repair Helicase XPD
Source: PLoS Biol. 2008 Jun 24;6(6):e149. doi: 10.1371/journal.pbio.0060149 (PMC2435149; doi:10.1371/journal.pbio.0060149)

A.

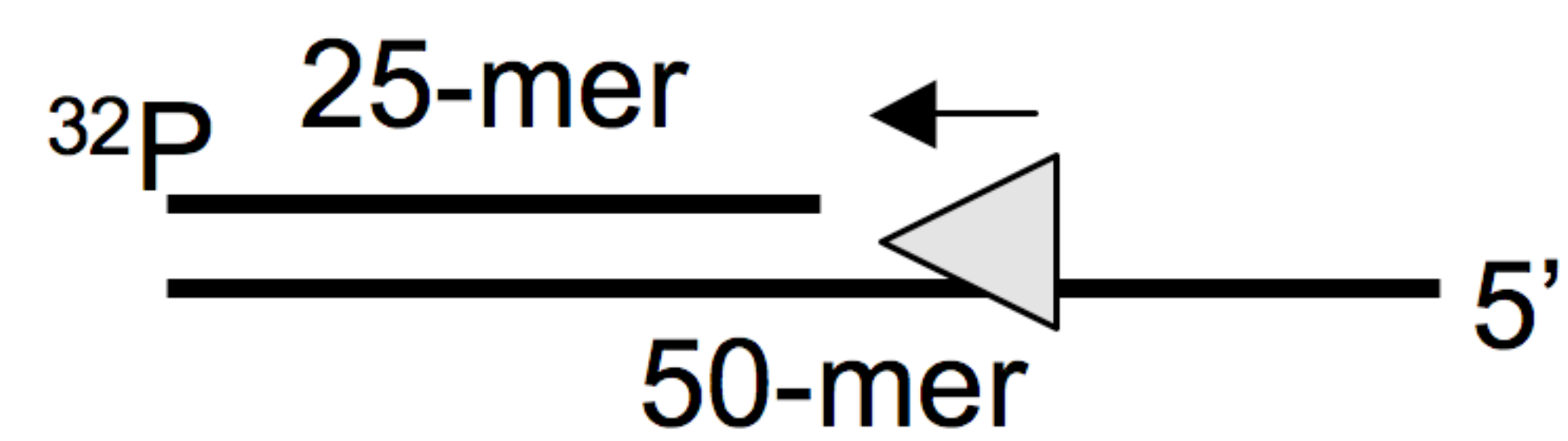

B.

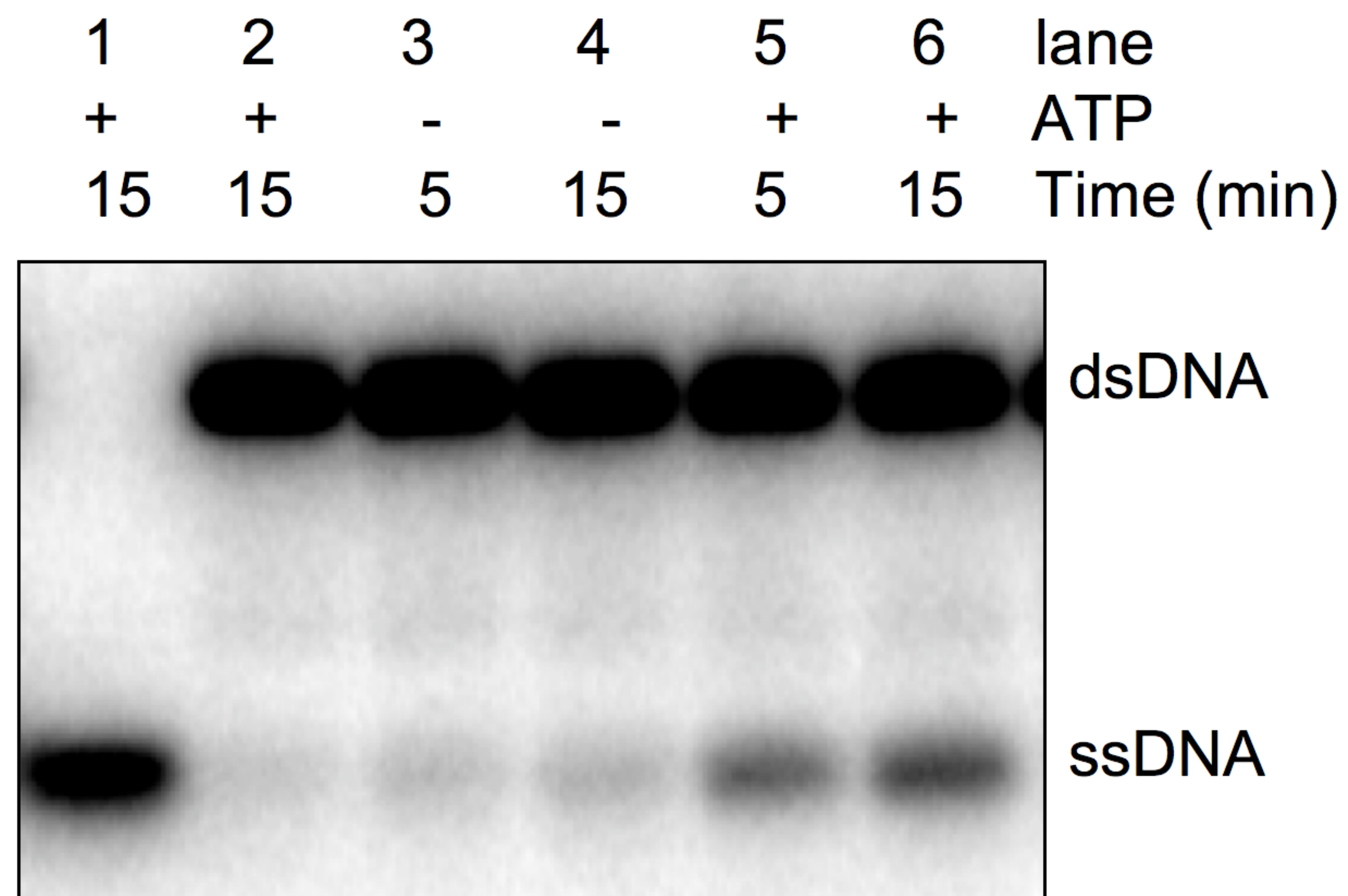

Supplemental Figure S1

Supplement: Figure S1 — (A) Graphic representation of helicase assay. (B) XPD is an ATP-dependent DNA helicase. Lane 1 is the ssDNA control, lane 2 dsDNA without XPD, lanes 3–6 contain 500 nM XPD in the absence (lanes 3 and 4) of ATP and in the presence of ATP (lanes 5 and 6). The 5-min and 15-min incubation times were analyzed and are shown in lanes 3 and 5 or 4 and 6, respectively. (322 KB PDF) [file pbio.0060149.sg001.pdf]

A

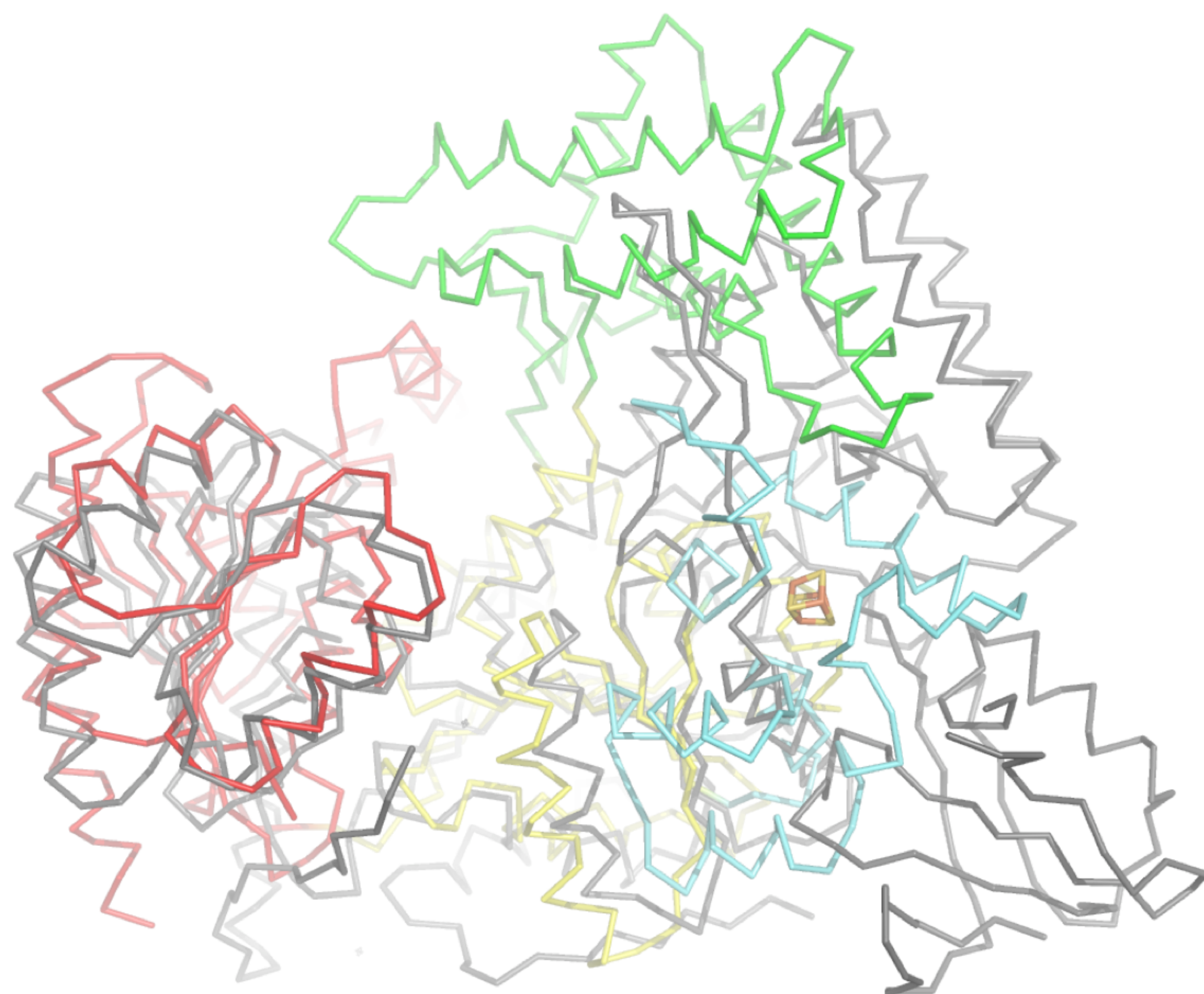

B

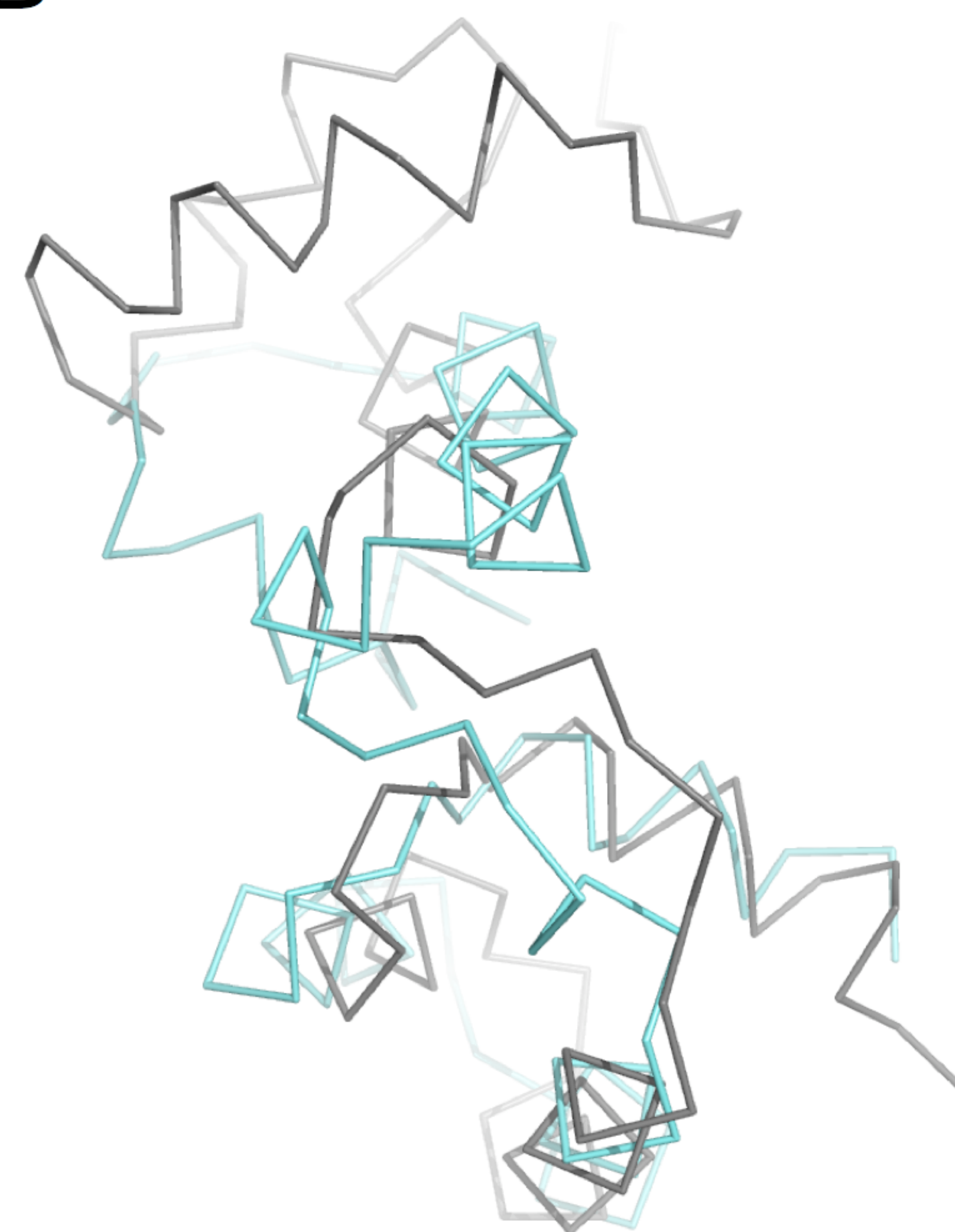

Supplemental Figure S2

Supplement: Figure S2 — (A) XPD, color coded as in Figure 1A, was superimposed with UvrB shown in grey. The two RecA-like domains (yellow and red) superimpose well, whereas the remainder of the two protein models share no structural homology. (B) Superposition of the 4Fe4S cluster containing domain of XPD with cMyb. XPD is shown in cyan and cMyb in grey. (959 KB PDF) [file pbio.0060149.sg002.pdf]

A

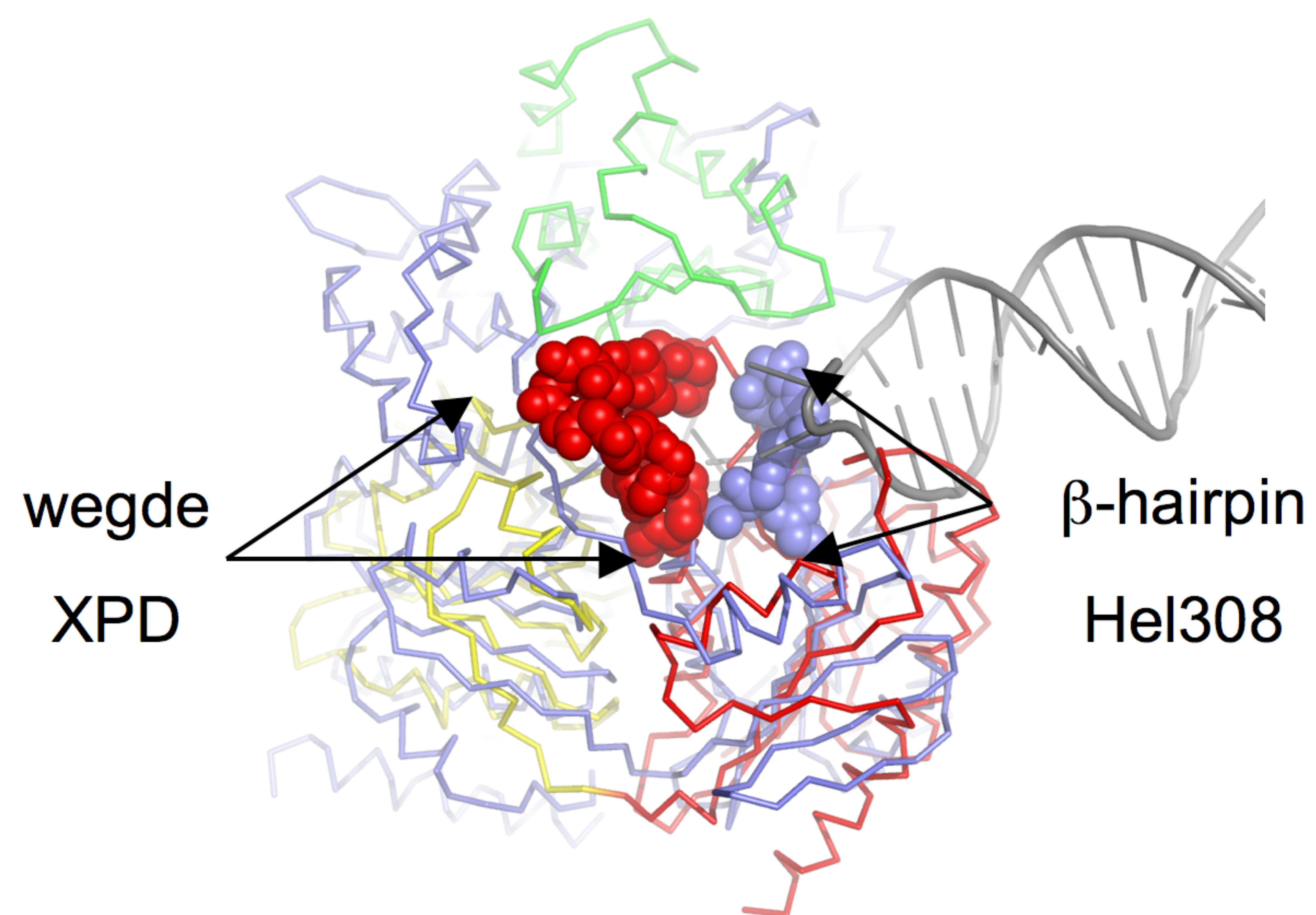

B

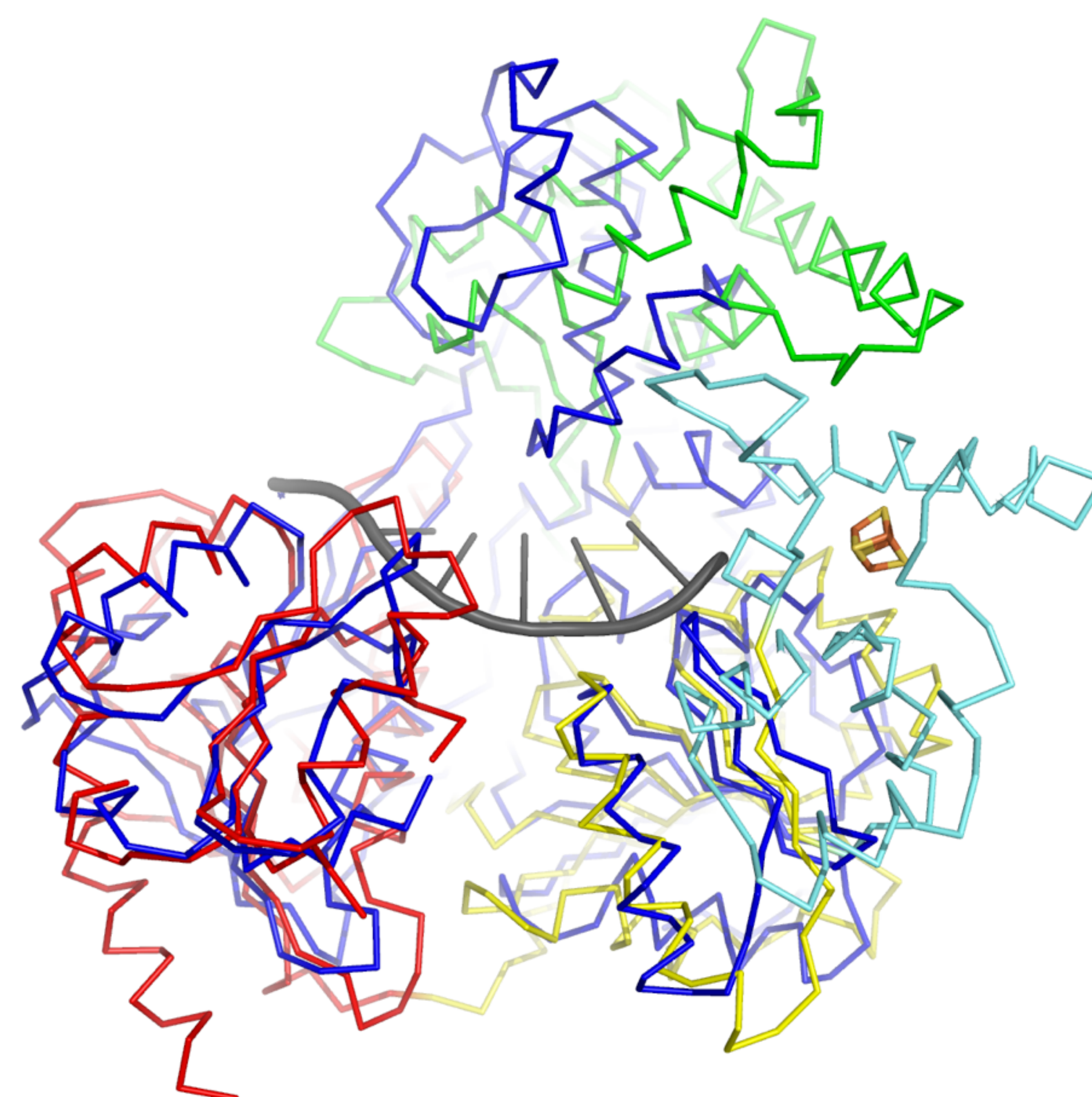

C

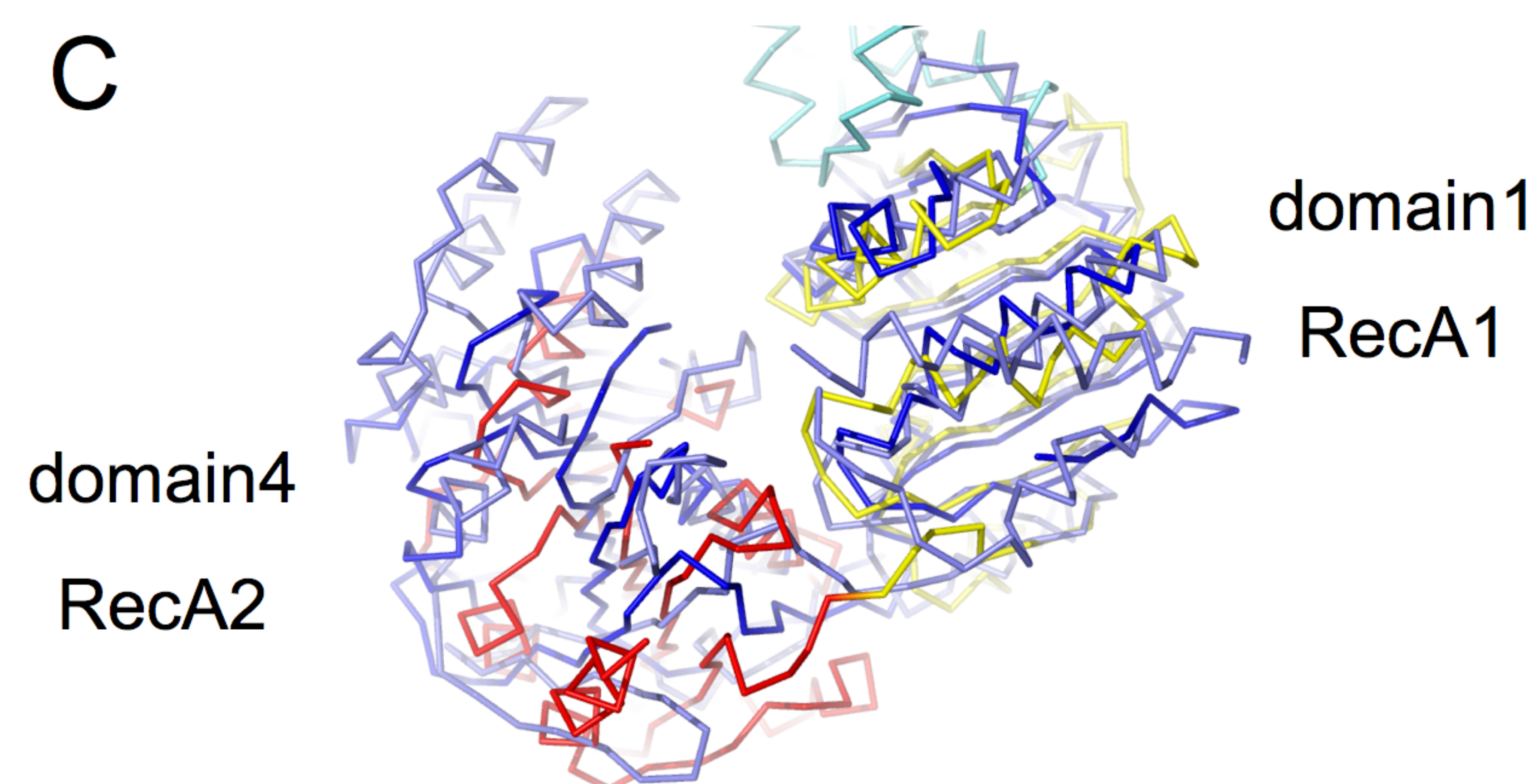

Supplemental Figure S3

Supplement: Figure S3 — (A) XPD is color coded as in Figure 1A; Hel 308 is shown in light blue. Main chain atoms of the wedge in XPD and the β-hairpin in Hel308 are shown in space-filling models and are indicated by arrows. The DNA is shown in grey, and the bases as grey spokes. The superposition of XPD with Hel308 revealed an rms deviation of 3.6 Å using 240 Cα-atoms out of 683 from Hel308 and 588 from XPD. (B) XPD is color coded as in (A), and NS3 is shown in blue. The superposition of NS3 helicase with XPD led to an rms deviation of 3.0 Å using 205 Cα-atoms out of 432 from NS3 and 588 from XPD. (C) Superpositions of the RecA-like domains of XPD (yellow and red), NS3 (blue), and Hel308 (light blue) using the first RecA domain (domain 1) as the pivot point. (1.59 MB PDF) [file pbio.0060149.sg003.pdf]
